# Simultaneous Determination and Pharmacokinetic Study of Six Components in Rat Plasma by HPLC-MS/MS after Oral Administration of *Acanthopanax sessiliflorus* Fruit Extract

**DOI:** 10.3390/ijms18010045

**Published:** 2016-12-28

**Authors:** Peng Du, Mingdao Lei, Yu Liu, Shilin Yang

**Affiliations:** 1 College of Pharmacy, Soochow University, Suzhou 215123, China; Dupeng_gyyxy@hotmail.com; 2 Drug Clinical Trial Institution, the Affiliated Hospital of Guizhou Medical University, Guiyang 550004, China; 3 Department of Pharmacy, Jiangxi Maternal and Child Health Hospital, No. 318 Bayi Road, Nanchang 330001, China; leimingdaojxnc@163.com; 4 College of Pharmacy, Liaoning University, Shenyang 110036, China; liuyu1981@163.com

**Keywords:** HPLC-MS/MS, *Acanthopanax sessiliflorus* fruits, rat plasma, pharmacokinetics

## Abstract

A specific and reliable HPLC-MS/MS method was developed and validated for the simultaneous determination of protocatechuic acid (PCA), scopolin, (−)-pinoresinol-4,4′-di-*O*-β-d-glucopyranoside (PDG), acanthoside D, acanthoside B and hyperin in rat plasma for the first time. The analytes were separated on a C_18_ column (50 × 2.1 mm, 1.8 µm) and a triple-quadrupole mass spectrometer equipped with an electrospray ionization (ESI) source was used for detection. The rat plasma sample was prepared using the protein precipitation procedure. The calibration curves were linear over a concentration range of 1.2–1200.0 ng/mL for PCA, 0.96–960.0 ng/mL for scopolin, 1.12–1120.0 ng/mL for PDG, 1.32–1320.0 ng/mL for acanthoside D, 0.99–990.0 ng/mL for acanthoside B and 1.01–1010.0 ng/mL for hyperin. The intra-day and inter-day precision was less than 11.4% and the relative error (RE) was all within ±15%. The validated method was successfully applied to assess the pharmacokinetics characteristics after the extracts of *Acanthopanax sessiliflorus* fruits were orally administered to the Sprague-Dawley rat.

## 1. Introduction

*Acanthopanax sessiliflorus* (*A. sessiliflorus*) Seem. is mainly distributed in China, Korea, and Japan. *A. sessiliflorus* fruits, a famous traditional Chinese medicine, has been reported to have antitumor and immunostimulating activities [1,2]. To use *A. sessiliflorus* fruits more efficiently and more conveniently, the main components of *A. sessiliflorus* fruits were extracted, such as protocatechuic acid (PCA), scopolin, (−)-pinoresinol-4,4’-di-*O*-β-d-glucopyranoside (PDG), acanthoside D, acanthoside B and hyperin [3,4,5]. Pharmacokinetic (PK) study of the active constituents in medicinal plants may be helpful to explain and predict a variety of events related to the efficacy and toxicity of Traditional Chinese Medicine (TCM). Therefore, to support the pharmacokinetic study of the extracts of *A. sessiliflorus* fruits, we wanted to develop a simple, sensitive method for the simultaneous determination of the six components mentioned above in biological fluids.

There have been some publications about the quantification of one or several components of *A. sessiliflorus* fruits in biological samples. The quantification of PCA in plasma by LC-MS/MS was extensively studied in many publications [6,7,8]. Xia et al. developed an LC-MS/MS method to determine scopolin in rat plasma [9,10]. Li et al. developed a LC-MS/MS method to determine hyperin in mouse plasma [11,12]. However, to the best of our knowledge, no other published reports are available with regard to the simultaneous quantification of PCA, scopolin, PDG, acanthoside D, acanthoside B and hyperin in plasma.

The aim of the present study was to develop and validate a selective and sensitive HPLC-MS/MS method for the simultaneous determination of the six components above in rat plasma and to investigate their pharmacokinetics in rats after the oral administration of the extracts of *A. sessiliflorus* fruits.

## 2. Results and Discussion

### 2.1. Method Development

**Figure 1:**
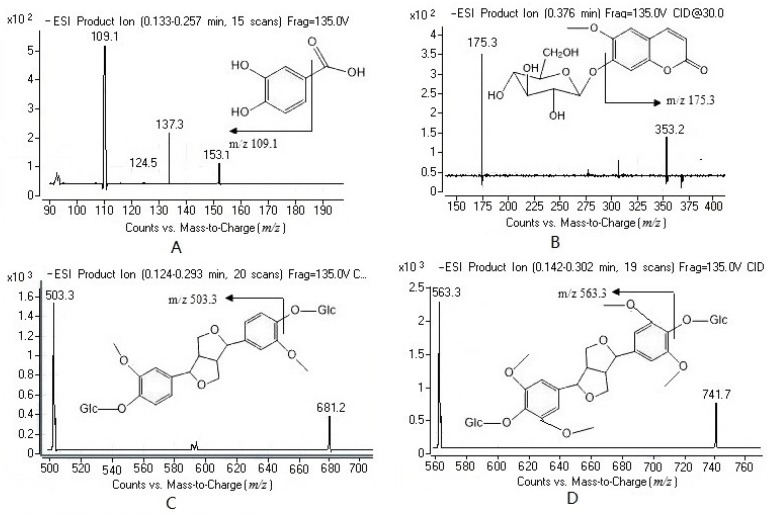

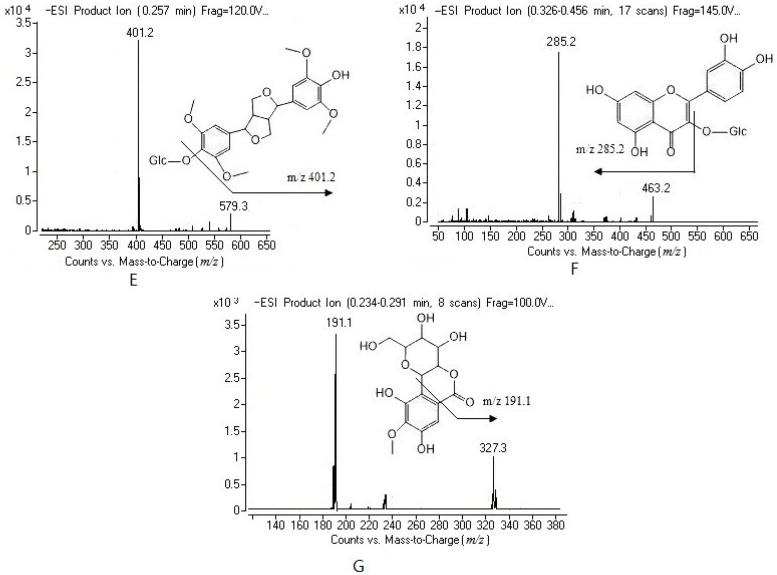
Product ion mass spectra of [M-H]^−^ ion of protocatechuic acid (PCA, **A**), scopolin (**B**), (−)-pinoresinol-4,4’-di-*O*-β-d-glucopyranoside (PDG, **C**), acanthoside D (**D**), acanthoside B (**E**), hyperin (**F**) and bergenin (**G**).

PCA had a carboxyl group in the molecular structure. It had a better response in the negative ESI mode than in the positive mode. The product ions of the [M–H]^−^ of PCA were dependent on the collision voltage. The major fragment ion *m*/*z* 137.3 was formed at the lower collision voltage, and the most abundant ion *m*/*z* 109.1 was formed at the higher collision voltage. The transition of *m*/*z* 153.1→109.1 gave a higher signal-to-noise (S/N) ratio and better response than that of *m*/*z* 153.1→137.3 during the analysis of the spiked plasma samples. As a result, the transition of *m*/*z* 153.1→109.1 was selected for MRM analysis of PCA (Figure 1). In the same optimization way, the transitions of 353.2→175.3, 681.2→503.3, 741.7→563.3, 579.3→401.2, 463.2→285.2, 327.3→191.1 were applied for the determination of scopolin, PDG, acanthoside D, acanthoside B, hyperin and bergenin, respectively. The addition of formic acid to the mobile phase can improve the peak shape, the ionization of the analytes and the mass response.

### 2.2. Method Validation

**Figure 2:**
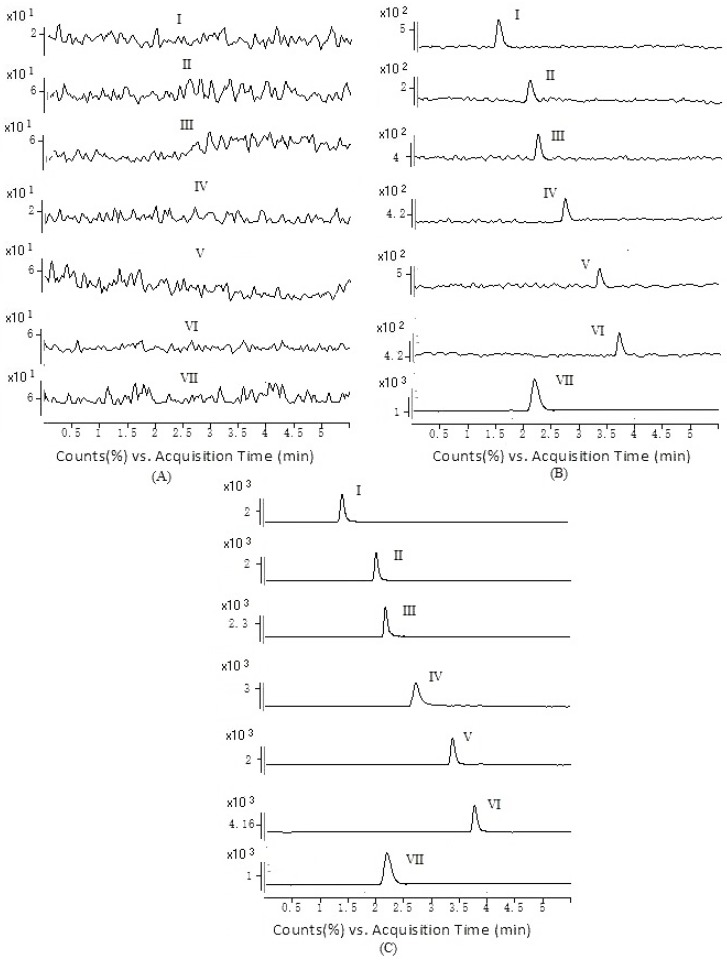
Representative MRM chromatograms of protocatechuic acid (I), scopolin (II), (−)-pinoresinol-4,4’-di-*O*-β-d-glucopyranoside (III), acanthoside D (IV), acanthoside B (V), hyperin (VI) and bergenin (I.S., VII) in rat plasma: (**A**) a blank rat plasma sample; (**B**) a spiked plasma sample with PCA (1.2 ng/mL), scopolin (0.96 ng/mL), PDG (1.12 ng/mL), acanthoside D (1.32 ng/mL), acanthoside B (0.99 ng/mL), hyperin (1.01 ng/mL) and the internal standard; (**C**) a rat plasma sample following an oral dose of the extracts of *A. sessiliflorus* fruits at 800 mg/kg (calculated as the total amount of extracts) to a Sprague-Dawley rat, PCA (176.2 ng/mL), scopolin (238.2 ng/mL), PDG (216.1 ng/mL), acanthoside D (123.3 ng/mL), acanthoside B (145.8 ng/mL), hyperin (19.4 ng/mL).

Figure 2 showed the typical chromatograms of a blank, a spiked plasma sample with PCA (1.2 ng/mL), scopolin (0.96 ng/mL), PDG (1.12 ng/mL), acanthoside D (1.32 ng/mL), acanthoside B (0.99 ng/mL), hyperin (1.01 ng/mL) and the internal standard, a plasma sample from a rat after oral administration of the extracts of *A. sessiliflorus* fruits. No interference from the endogenous compound with the analytes and the internal standard was found.

The linear regressions of the peak area ratios versus concentration were fitted over the concentration range of 1.2–1200.0 ng/mL for PCA, 0.96–960.0 ng/mL for scopolin, 1.12–1120.0 ng/mL for PDG, 1.32–1320.0 ng/mL for acanthoside D, 0.99–990.0 ng/mL for acanthoside B and 1.01–1010.0 ng/mL for hyperin in rat plasma. The typical equation of the calibration curves was as follows: PCA: *y* = 0.0034*x* + 0.0123, *r* = 0.991; scopolin, *y* = 0.0018*x* + 0.0067, *r* = 0.994; PDG: *y* = 0.0371*x* + 0.0702, *r* = 0.993; acanthoside D: *y* = 0.0071*x* + 0.0302, *r* = 0.998; acanthoside B: *y* = 0.0048*x* + 0.0602, *r* = 0.997; hyperin: *y* = 0.0052*x* + 0.0491, *r* = 0.995, where *y* represents the peak area ratio of analytes to IS and *x* represents the concentration of the analytes in plasma. The correlation coefficient (*r*) exceeded 0.99, showing a good linearity among the concentration ranges.

The lower limit of quantification (LLOQ) was 1.2 ng/mL for PCA, 0.96 ng/mL for scopolin, 1.12 ng/mL for PDG, 1.32 ng/mL for acanthoside D, 0.99 ng/mL for acanthoside B and 1.01 ng/mL for hyperin The concentration of PCA, scopolin, PDG, acanthoside D, acanthoside B and hyperin can be determined for more than 8 h with the present LLOQ, which allowed for the investigation of the pharmacokinetics of the extracts of A. sessiliflorus fruits after oral administration. The precision and accuracy of six analytes at LLOQ level were all within the accepted limits. 

The matrix effects calculated were in the range of 92.4%–107.8%. Therefore, ion suppression or enhancement from rat plasma was negligible under the current conditions.

Table 1 summarizes the intra- and inter-day precision and accuracy for the analytes in QC samples. The intra- and inter-day RSD were below 11.4%, and the relative errors were from −3.9% to 10.9%. All the values were within the accepted range and the method was accurate and precise.

Six analytes were stable after three complete freeze/thaw cycles (−40 to 23 °C), long-term sample storage (−40 °C for 30 days) and bench-top (23 °C for 2 h). The extracted samples on the autosampler rack at 4 °C were stable for 6 h (Table 2).

### 2.3. Application to the PK Study in the Sprague–Dawley Rats

**Figure 3:**
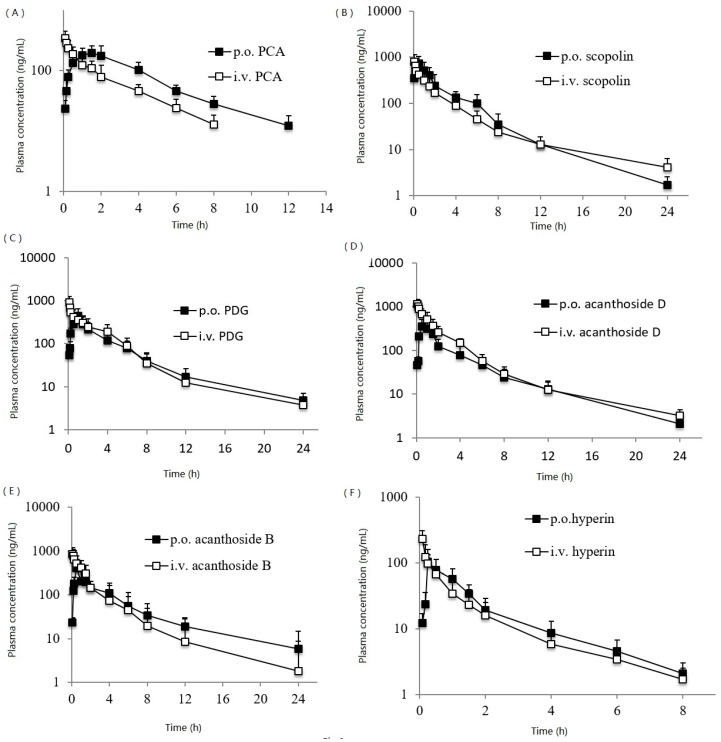
Plasma concentration–time profiles in the Sprague–Dawley rat. (■): oral administration of the extracts of *A. sessiliflorus* fruits to the Sprague–Dawley rat at 800 mg/kg (calculated as the total amount of extracts), (□): intravenous injection route. p.o.: per oral, i.v: intravenous injection.

This validated method was applied to the PK studies after the oral administration of the extracts of *A. sessiliflorus* fruits to the Sprague–Dawley rat. The typical plasma concentration–time profiles of PCA, scopolin, PDG, acanthoside D, acanthoside B and hyperin are shown in Figure 3. The PK parameters are listed in Table 3. The *t*_max_ of six components were from 0.25 to 1.5 h, which indicated the absorption of the components was relatively fast. The t_1/2_ were from 0.89 to 2.34 h, which showed all components were metabolized in vivo with high speed. The oral bioavailability was from 5.1% to 25.4%, which was relatively low.

## 3. Experimental

### 3.1. Chemicals and Reagents

PCA (purity > 99%) and bergenin (IS, purity > 99%) were purchased from the National Institute for the Control of Pharmaceutical and Biological Products (Beijing, China). Scopolin, PDG, hyperin (purity > 99%) were purchased from Chengdu Beisite Reagents Corp. (Chengdu, China). Acanthoside D (purity > 99%) was provided by Shenyang Pharmaceutical University (Shenyang, China). Acanthoside B (purity > 99%) was purchased from Shanghai Fengshou Biotechnology Corp. (Shanghai, China). Methanol and acetonitrile was purchased from Fisher Scientific (Pittsburgh, PA, USA). Formic acid (FA) was purchased from Sigma-Aldrich (St. Louis, MO, USA). Water was prepared using a Mill-Q ultrapure water system (Millipore Corp., Billerica, MA, USA). All other chemicals were of analytical grade.

For preparation of the extracts, *A. sessiliflorus* fruits (200 g) was extracted with 1600 mL water-presaturated *n*-butanol for 3 times at 60 °C, 30 min each time, and then filtrated. The combined filtrate was evaporated to dryness. The content of PCA, scopolin, PDG, acanthoside D, acanthoside B and hyperin in the extracts was 8.9%, 3.5%, 6.7%, 5.6%, 9.1% and 13.3%, respectively.

### 3.2. Instrumentation

An Agilent 1290 ultra-performance liquid chromatography and an Agilent 6460 triple-quadrupole tandem mass spectrometer (Agilent Technologies, Santa Clara, CA, USA) were used for the determination of the analytes. An Agilent ZORBAX SB-C18 column (50 × 2.1 mm, 1.8 µm) was used to separate the analytes. All data were acquired and processed using Masshunter^TM^ 2.0 software (Agilent Technologies, Santa Clara, CA, USA).

### 3.3. HPLC/MS/MS Conditions

A gradient elution program was conducted for chromatographic separation with the mobile phase A (methanol containing 0.1% formic acid), and the mobile phase B (water containing 0.1% formic acid) as follows: 0 min (11%, A), 2 min (25%, A), 3.5 min (75%, A), 4.0 min (11%, A), 5.5 min (11%, A). The flow rate was 0.2 mL/min and the column temperature was 30 °C.

The mass spectrometry was operated in the negative mode. Quantification was performed by multiple reaction monitoring (MRM). N_2_ (purity of 99.9%) was used as drying gas (13 L/min) and nebulizing gas (40 L/min). Gas temperature was 350 °C. Capillary voltage was 4300 V. The MRM transition of 153.1→109.1, 353.2→175.3, 681.2→503.3, 741.7→563.3, 579.3→401.2, 463.2→285.2, 327.3→191.1 was applied for the determination of PCA, scopolin, PDG, acanthoside D, acanthoside B, hyperin, and bergenin, respectively. N_2_ (purity of 99.999%) was used as collision gas, and the dwell time was 60 ms.

During the optimization of the MRM transitions, the standard solution was infused into MS spectrometry via the needle of the sampler system in HPLC at 200 µL/min. The concentrations were 120.0 ng/mL for PCA, 96.0 ng/mL for scopolin, 112.0 ng/mL for PDG, 132.0 ng/mL for acanthoside D, 99.0 ng/mL for acanthoside B, 101.0 ng/mL for hyperin, and 167.0 ng/mL for bergenin, respectively.

### 3.4. Preparation of Standard and Quality Control Samples

The stock mixing standard solutions of PCA, scopolin, PDG, acanthoside D, acanthoside B and hyperin was prepared in methanol at concentrations of 1.20, 0.96, 1.12, 1.32, 0.99, and 1.01 µg/mL, respectively. The working solution of the desired concentration was prepared by diluting serially the stock solution with methanol. The internal standard solution at the 1.67 µg/mL level was prepared in methanol. The calibration curves with seven non-zero standard levels consisted of PCA/scopolin/PDG/acanthoside D/acanthoside B /hyperin in the concentration of 1.2–1200.0 ng/mL for PCA, 0.96–960.0 ng/mL for scopolin, 1.12–1120.0 ng/mL for PDG, 1.32–1320.0 ng/mL for acanthoside D, 0.99–990.0 ng/mL for acanthoside B and 1.01–1010.0 ng/mL for hyperin. The calibration curves were prepared by spiking 50 µL of blank rat plasma with 50 µL of the mixing standard solution of PCA/scopolin/PDG/acanthoside D/acanthoside B/hyperin, 40 μL of of internal standard solution. The quality control (QCs) samples at low, medium, high concentration levels were prepared in the same way as the calibration curves. The nominal plasma concentrations of QC samples were PCA (2.4, 600.0, 960.0 ng/mL), scopolin (1.92, 480.0, 768.0 ng/mL), PDG (2.24, 560.0, 896.0 ng/mL), acanthoside D (2.64, 660.0, 1056.0 ng/mL), acanthoside B (1.98, 495.0, 960.0 ng/mL), hyperin (2.02, 510.0, 808.0 ng/mL). All solutions were stored at −40 °C.

### 3.5. Sample Preparation

To a 50-µL aliquot of plasma sample, 50-μL methanol, 40-μL IS solution and 40-μL water containing 0.1% formic acid were added. The sample was briefly vortex-mixed for 1 min. Then 300-μL methanol was added and mixed for 5 min. The sample was centrifuged at 15,000 rpm for 10 min to precipitate the protein. The supernatant was collected and evaporated to dryness by N_2_ at 37 °C. Finally, the residue was dissolved in 150-μL methanol. A 5-μL aliquot was injected for HPLC-MS/MS analysis.

### 3.6. Method Validation

The method was fully validated in terms of selectivity, linearity, LLOQ, accuracy and precision, extraction recovery and matrix effect, and stability according to FDA guidelines for the validation of the bioanalytical method [13].

Selectivity was investigated by comparing chromatograms of blank rat plasma from six different batches with the corresponding spiked rat plasma. Linearity was assessed by weighted (1/x2) least-squares analysis of six different calibration curves. The intra- and inter-day precision (the relative standard deviation, RSD) and accuracy (the relative error, RE) were evaluated by performing the determination of QC samples at three concentration levels (low, medium, and high) in six replicates over three separate days. The matrix effect (ME) was assessed by comparing the peak areas of analytes in the post-extraction spiked blank plasma at low and high concentrations with those of the corresponding standard solutions. The extraction recovery was determined by comparing the mean peak areas of the regularly pretreated QC samples at low, medium, and high concentrations with the mean peak areas of spike-after-extraction samples. The stability of analytes in rat plasma was investigated by analyzing triplicate samples at low and high QC levels after the spiked samples were exposed to room temperature (23 °C) for 2 h and the ready-to-inject samples in an autosampler were exposed to 4 °C for 6 h. The stability of low and high QC samples (*n* = 3) in three complete freeze/thaw cycles (−40 to 23 °C), long-term sample storage (−40 °C for 30 days) was also assessed.

### 3.7. Pharmacokinetic Study

Male Sprague–Dawley rats weighing from 200 to 240 g were used for the PK study. All animal experiments were performed in accordance with institutional guidelines and were approved by the University Committee on Use and Care of Animals, Soochow University. The rats were kept in an animal room with a constant temperature (20 ± 2 °C), relative humanity (50% ± 10%) and a 12 h light/dark cycle. Forty-two rats were fasted overnight with free access to water prior to the experiment and were randomly assigned into 7 groups. The first group was orally administered with the suspension of extracts of *A. sessiliflorus* fruits at 800 mg/kg (calculated as the total amount of extracts, equivalent to PCA 71.2 mg/kg, scopolin 28.0 mg/kg, PDG 53.6 mg/kg, acanthoside D 44.8 mg/kg, acanthoside B 72.8 mg/kg and hyperin 106.4 mg/kg), and the other six groups were injected with PCA, scopolin, PDG, acanthoside D, acanthoside B and hyperin (all at 5 mg/kg, the injection solution was prepared with ethanol-water (10:90)) via tail vein, respectively. Blood samples of approximately 200-μL were collected from the orbital vein at pre-dosing and 5, 10, 15, 30 min and 1, 1.5, 2, 4, 6, 8, 12, 24 h post-dosing. Blood samples were placed in heparin-containing tubes and immediately centrifuged at 13,000 × *g* for 5 min at 4 °C. Plasma was collected and frozen at −40 °C until analysis.

## 4. Conclusions

The present study developed and validated a facile, specific, and sensitive HPLC-MS/MS method for the simultaneous determination of PCA, scopolin, PDG, acanthoside D, acanthoside B and hyperin in rat plasma. The analysis method was very convenient because of the simple sample pretreatment and the short analysis run time. This method has been successfully applied to PK studies after oral administration of the extracts of *A. sessiliflorus* fruits to Sprague–Dawley rats.

## Figures and Tables

**Table 1:** Precision and accuracy of six components in rat plasma (in three validation days, six replicates at different concentration levels per day).

Concentration (ng/mL)		RSD (%)		RE (%)
Added	Found (Mean)	Intra-Day	Inter-Day	
PCA				
2.40	2.61	8.7	9.3	8.8
600.0	643.6	6.5	7.4	7.3
960.0	998.5	6.1	5.2	4.0
Scopolin				
1.92	2.02	7.5	6.3	5.2
480.0	510.2	5.1	8.2	6.3
768.0	805.1	3.7	5.5	4.8
PDG				
2.24	2.41	10.2	9.9	7.6
560.0	583.8	6.8	8.3	4.2
896.0	928.5	4.7	4.1	3.6
Acanthoside D				
2.64	2.87	10.1	11.4	8.7
660.0	634.5	5.3	6.4	−3.9
1056.0	1123.6	4.6	5.9	6.4
Acanthoside B				
1.98	2.12	7.7	9.1	7.1
495.0	523.4	8.1	6.4	5.7
960.0	1003.2	3.9	4.7	4.5
Hyperin				
2.02	2.24	9.3	7.4	10.9
510.0	543.7	8.1	8.5	6.6
808.0	845.9	5.9	5.2	4.7

The mean extraction recoveries were 87.3% ± 5.6%, 90.2% ± 6.3%, 82.5% ± 4.3%, 85.4% ± 4.8%, 93.7% ± 5.7%, 97.3% ± 4.7% for PCA, scopolin, PDG, acanthoside D, acanthoside B and hyperin, respectively. The mean recovery of the internal standard was 78.4% ± 6.1%.

**Table 2:** Stability data of six components in rat plasma under different conditions.

Conditions	Concentration (ng/mL)		RSD (%)	RE (%)
	Added	Found (Mean)		
Bench-Top (23 °C for 2 h)				
PCA	2.40	2.54	9.4	5.8
	960.0	993.2	6.7	3.5
Scopolin	1.92	2.04	10.3	6.3
	768.0	798.2	5.3	3.9
PDG	2.24	2.45	8.5	9.4
	896.0	934.7	4.9	4.3
Acanthoside D	2.64	2.45	8.2	−7.2
	1056.0	1103.2	3.7	4.5
Acanthoside B	1.98	2.12	9.3	7.1
	960.0	989.5	6.4	3.1
Hyperin	2.02	2.19	11.3	8.4
	808.0	843.8	6.2	4.4
Three freeze/thaw cycles(−40 to 23 °C)				
PCA	2.40	2.56	8.8	6.7
	960.0	988.4	6.1	3.0
Scopolin	1.92	2.08	9.5	8.3
	768.0	803.2	5.7	4.6
PDG	2.24	2.41	10.3	7.6
	896.0	932.1	4.9	4.0
Acanthoside D	2.64	2.87	7.9	8.7
	1056.0	1098.5	7.1	4.0
Acanthoside B	1.98	1.78	11.5	−10.1
	960.0	904.6	6.2	−5.8
Hyperin	2.02	2.14	7.6	5.9
	808.0	832.6	5.2	3.0
Autosampler rack at 4 °C for 6 h				
PCA	2.40	2.19	8.3	−8.8
	960.0	998.4	4.8	4.0
Scopolin	1.92	1.78	8.1	−7.3
	768.0	813.5	6.2	5.9
PDG	2.24	2.41	10.7	7.6
	896.0	934.2	4.9	4.3
Acanthoside D	2.64	2.87	9.8	8.7
	1056.0	1098.5	5.4	4.0
Acanthoside B	1.98	2.18	9.7	10.1
	960.0	1004.2	9.4	4.6
Hyperin	2.02	2.14	10.3	5.9
	808.0	845.6	6.9	4.7
Freezing at −40 °C for 30 days				
PCA	2.40	2.59	7.3	7.9
	960.0	993.6	8.1	3.5
Scopolin	1.92	2.04	8.5	6.3
	768.0	804.3	6.2	4.7
PDG	2.24	2.37	9.4	5.8
	896.0	923.7	7.5	3.1
Acanthoside D	2.64	2.88	11.3	9.1
	1056.0	1089.4	9.2	3.2
Acanthoside B	1.98	2.15	9.6	8.6
	960.0	1003.8	4.7	4.6
Hyperin	2.02	2.19	8.2	8.4
	808.0	843.8	6.1	4.4

**Table 3:** Mean pharmacokinetic parameters for Protocatechuic acid (PCA), Scopolin, (−)-pinoresinol-4,4’-di-*O*-β-d-glucopyranoside (PDG), Acanthoside D, Acanthoside B and Hyperin in rat plasma after the oral administration of the extracts of *Acanthopanax sessiliflorus* fruits at 800 mg/kg (calculated as the total amount of extracts, equivalent to PCA 71.2 mg/kg, scopolin 28.0 mg/kg, PDG 53.6 mg/kg, acanthoside D 44.8 mg/kg, acanthoside B 72.8 mg/kg and hyperin 106.4 mg/kg).

Compound	*C*_max_ (ng/mL)	*t*_max_ (h)	*t*_1/2_ (h)	AUC_0–∞_ (ng·h/mL)	F (%)
PCA	198.1 ± 60.3	1.5 ± 0.5	1.0 ± 0.2	901.3 ± 132.1	11.9
Scopolin	734.1 ± 203.4	0.5 ± 0.3	1.4 ± 0.5	1876.9 ± 398.4	25.4
PDG	447.1 ± 104.5	1.0 ± 0.4	2.3 ± 0.8	1470.9 ± 350.5	7.7
Acanthoside D	356.7 ± 145.2	0.5 ± 0.3	2.0 ± 0.6	1042.0 ± 285.1	6.0
Acanthoside B	409.5 ± 133.2	0.5 ± 0.2	1.2 ± 1.0	1216.9 ± 315.0	6.2
Hyperin	103.7 ± 46.3	0.25 ± 0.3	0.9 ± 0.5	151.0 ± 26.1	5.1

AUC for PCA, Scopolin, PDG, Acanthoside D, Acanthoside B and Hyperin after intravenous injection at 5 mg/kg was 529.8 ± 103.2, 1313.7 ± 345.6, 1791.2 ± 509.8, 1941.8 ± 443.1, 1355.5 ± 302.6 and 140.3 ± 45.1 ng·h/mL, respectively.
